# Sampling-Age-Associated Cecal Community and Intestinal Transcriptional Responses to Dietary Astragalus Polysaccharides in Taihu Geese

**DOI:** 10.3390/microorganisms14102149

**Published:** 2026-09-23

**Authors:** Xiaoming Li, Mengli Zhao, Lei Zhang, Guobo Sun, Xiujun Duan, Guoshun Chen

**Affiliations:** 1 The Animal Science and Technology College, Gansu Agricultural University, Lanzhou 730070, China; 2 The Animal Science and Technology College, Jiangsu Agri-Animal Husbandry Vocational College, Taizhou 225300, China; 3 National Gene Bank of Waterfowl Resources, Taizhou 225300, China

**Keywords:** Astragalus polysaccharides, Taihu goose, cecal microbiota, intestinal transcriptome, 16S rRNA sequencing, RNA sequencing

## Abstract

Sampling age-associated microbial and intestinal transcriptional responses to Astragalus polysaccharides (APS) remain unclear in geese. In a 90-day experiment, 150 one-day-old Taihu geese received 0, 100, or 200 mg/kg APS, with five replicate cages per diet. Cecal 16S rRNA gene profiles were obtained at days 30, 60, and 90 (n = 4 per diet and age); ileal and jejunal RNA sequencing was performed at day 90 (n = 4 per diet and tissue). Sampling age explained 15.1% of the Bray–Curtis variation (q = 0.0012), APS explained 6.7% after age adjustment (q = 0.0144), and their interaction was significant (q = 0.0128). Community associations were stable across amplicon sequence variant filtering thresholds; no APS association with alpha diversity or an individual genus survived correction. For 100 and 200 mg/kg APS, respectively, 43 and three ileal genes and nine and seven jejunal genes met q < 0.05 and |log_2_ fold change| ≥ 1. Jejunal expression was associated with RNA integrity, limiting transcriptomic interpretation. Chicken single-cell markers contextualized part of the ileal signature at 100 mg/kg APS; no diet-adjusted paired microbiome–pathway association survived correction. APS-associated community and transcriptional patterns remain exploratory within the phase-specific feeding program.

## 1. Introduction

The gastrointestinal tract of geese supports nutrient digestion and extensive hindgut fermentation. However, its microbial ecology and mucosal responses require species-specific investigation. Host genomic and metagenomic analyses have linked carbohydrate and energy metabolism to goose–microbiome adaptation [1]. Additionally, microbial composition and short-chain fatty acid concentrations vary substantially among gastrointestinal segments [2], while cecal community structure changes throughout post-hatch development [3]. Age and intestinal compartment are essential design factors when evaluating feed additives in geese.

Astragalus polysaccharides (APS) are heterogeneous carbohydrate fractions isolated from Astragalus species. Their specific composition and biological activity vary based on several factors. These include the plant source, extraction and purification procedures, molecular-weight distribution, branching, and contaminant control. In poultry, dietary APS has been associated with production traits, inflammatory and antioxidant indices, intestinal morphology, and gut microbial composition [4,5,6]. Furthermore, APS-related microbial shifts have been linked to intestinal barrier function in broilers [6]. However, it remains unresolved whether Taihu geese exhibit comparable responses. It is also unknown how these responses vary with sampling age, dose, or specific intestinal regions.

Rapid microbiome maturation can obscure treatment-by-sampling-age responses when observations are pooled across time. In addition, taxon-wise testing in a small cohort increases the risk of false-positive findings unless multiplicity is controlled. Cross-omics correlations may arise from treatment-group structure or mismatched samples rather than from biological covariation between matched microbial and host measurements across animals. Consequently, reliable paired analysis requires animal-level identifiers, diet adjustment, restricted permutations, and appropriately bounded interpretation.

This study integrated sampling age-resolved cecal 16S rRNA profiling with ileal and jejunal bulk RNA sequencing at 90 days. Wing number matching enabled within-animal multi-omics analysis, while an independent chicken ileal single-cell dataset supplied cell marker context. We hypothesized that APS-associated changes in cecal community structure would differ among sampling ages and that intestinal transcriptional responses would vary by APS dose and intestinal segment. As an explicitly exploratory objective, we also investigated whether matched microbial and host pathway measurements at 90 days exhibited diet-adjusted covariation across animals. Because the basal formulation changed after day 30, sampling age was interpreted within the phase-specific feeding program rather than as an isolated chronological age effect.

## 2. Materials and Methods

### 2.1. Animals, Experimental Design, Diets, and Ethics

A total of 150 healthy one-day-old Taihu geese were obtained from the National Gene Bank of Waterfowl Resources in Taizhou, China. The mean initial body weight was 90.26 ± 0.31 g. The birds were randomly assigned to a control diet (CON; basal diet without APS), a basal diet supplemented with 100 mg/kg APS (APS100), or a basal diet supplemented with 200 mg/kg APS (APS200). Each treatment comprised five replicate cages of 10 birds, with five males and five females per cage. Replicate groups were housed in separate cages, and the cage was the experimental unit for the feeding trial. The 90-day feeding period comprised days 1–30, 31–60, and 61–90.

APS was supplied as a commercial finished preparation by Macklin Biochemical (Shanghai, China; CAS 89250-26-0; catalog no. C832678). The supplier-reported polysaccharide content was at least 70.0%. Nominal APS supplementation levels of 100 and 200 mg/kg diet were expressed on a polysaccharide content basis and calculated using 70.0% as the formulation conversion factor, equivalent to approximately 142.9 and 285.7 mg of the commercial preparation per kilogram of diet, respectively. APS was incorporated into the ground basal diet by serial mixing, and all diets were provided in mash form. The 100 and 200 mg/kg supplementation levels followed the original feeding protocol and represented two practical low-dose levels within the 100–400 mg/kg range previously evaluated in poultry [4]. The two supplementation levels were included to compare responses across the tested dose range.

Birds were housed in their assigned single-tier replicate cages, with 10 birds per cage at the start of the experiment. Room temperature was maintained at 27–29 °C on days 1–5, 25–27 °C on days 6–10, 22–25 °C on days 11–15, 19 °C on days 16–20, and approximately 18 °C thereafter. Feed and water were provided ad libitum. Before the experiment, the housing facility was cleaned and disinfected. Additionally, all birds were vaccinated and individually numbered. Diet 802 was fed on days 1–30, and diet YH was fed on days 31–90. Retained samples from the same basal diet batches used in this experiment were received by the Central Laboratory of Anyou Biotechnology Group on 25 February 2026, and analysis was completed on 4 March 2026. Moisture, crude protein, crude ash, calcium, total phosphorus, crude fiber, and amino acids were determined using GB/T 6435-2014, GB/T 6432-2018, GB/T 6438-2025, GB/T 6436-2018, GB/T 6437-2018, GB/T 6434-2022, and GB/T 18246-2019, respectively. The basal diet ingredients and selected calculated and analyzed nutrient levels are presented in Table 1.

At 30, 60, and 90 days, 12 birds from each dietary group were selected after a 12 h feed withdrawal. The selection favored birds with body weights close to the corresponding cage mean. The omics subset comprised four birds per diet and sampling age, identified by wing number. However, the archived omics manifest did not retain the sex or direct cage identifier of the sequenced birds. Consequently, the sex distribution and cage allocation of the omics subset could not be reconstructed, and neither factor was included in the omics models. Sampling-time individual body weights were also not retained in the linked omics manifest. Wing numbers were used to match specimens across omics layers and were not treated as a substitute for missing body weight records. The underlying longitudinal cage-level feed-consumption data, longitudinal body weight records, and sample-level sex linkage required for valid treatment- or sex-stratified analyses of growth performance were not available in the analytic archive. Accordingly, growth performance was outside the scope of the present omics study.

Each selected goose was stunned and killed individually by personnel trained in animal handling and poultry stunning. Head-only electrical stunning was performed using an ASSOM-ELEC P-LINK poultry stunning system fitted with an electrode-holder handle (ref. 4018-5; BIOLINK System, Saint-Étienne-de-Tulmont, France; supplied by Burdis SARL, La Fouillouse, France), followed by ventral neck cutting and exsanguination. Head-only electrical stunning was followed by ventral neck cutting and exsanguination. Unconsciousness was confirmed before neck cutting, and death was confirmed before tissue collection. Any ineffective stun or sign of recovery triggered immediate restunning with a checked backup device. All procedures followed institutional SOP JSAHVC-SOP-GOOSE-001, Version 1.0. Details of electrode placement, electrical settings, monitoring criteria, time limits, rescue procedures, and the complete death-confirmation checklist are provided in Supplementary Methods S1.

All specimens and data analyzed in this study originated from the experiment approved by the Animal Ethics Committee of Jiangsu Agri-Animal Husbandry Vocational College on 15 February 2024. The animal facility operated under license SYXK (Su) 2012-0029. No additional live-animal procedures were conducted for the analyses reported in this study.

### 2.2. Cecal 16S rRNA Gene Sequencing and Bioinformatic Analysis

Cecal content (2–3 g) was collected at 30, 60, and 90 days from four birds per diet and sampling age. Samples were immediately frozen in liquid nitrogen and stored at −80 °C. The resulting dataset comprised 36 libraries, each linked to an individual wing number.

Genomic DNA was extracted using a DP812 kit (Tiangen Biotech, Beijing, China). DNA quantity and integrity were assessed using a NanoDrop 2000 spectrophotometer (Thermo Fisher Scientific, Waltham, MA, USA) and agarose gel electrophoresis. The bacterial 16S rRNA V3–V4 region was amplified with the barcoded primers 343F (5′-TACGGRAGGCAGCAG-3′) and 798R (5′-AGGGTATCTAATCCT-3′) using TaKaRa Ex Taq DNA Polymerase (catalog no. RR001A; Takara Biotechnology (Dalian) Co., Ltd., Dalian, China). Amplicons were verified by agarose gel electrophoresis, purified with AMPure XP beads (Beckman Coulter, Inc., Brea, CA, USA; lot no. 21483100), and quantified using a Qubit 4 Fluorometer (catalog no. Q33226; Thermo Fisher Scientific, Waltham, MA, USA). OE Biotech (Shanghai, China) sequenced the libraries on an NovaSeq 6000 Sequencing System (Illumina, Inc., San Diego, CA, USA) to generate 250 bp paired-end reads. Cutadapt version 4.4 was used to remove primer sequences [7]. Quality filtering, denoising, paired-read merging, and chimera removal were performed using q2-dada2 plugin under the default settings of QIIME 2 version 2020.11 [8,9]. Taxonomy was assigned to amplicon sequence variant (ASV) representative sequences against the SILVA database (release 138) using the q2-feature-classifier plugin in QIIME 2 version 2020.11 with default settings [10]. ASV counts and taxonomy tables were generated under the vendor project DZOE2025010373. This project included all 36 libraries, allocating four libraries to each diet-by-sampling-age cell. The archived metadata lacked separate extraction, library-preparation, lane, or sequencing batch variables. Consequently, no technical batch covariate or cage-by-batch allocation could be reconstructed. The raw amplicon datasets are publicly available in the NCBI Sequence Read Archive under BioProject PRJNA1504520 (SRA study SRP722618; runs SRR39911570–SRR39911605).

Only features assigned to Bacteria were retained. Chloroplast and mitochondrial assignments were removed. ASVs were required to have at least 50 reads across the complete 36-sample dataset and to occur in at least two samples. This criterion was based on cumulative read counts across the dataset and was not applied as a per-sample read threshold. This pragmatic filter removed sparse or single-sample features that would otherwise contribute unstable zeros to distance- and genus-level analyses. To evaluate the sensitivity of this filter, alternative thresholds of 10 and 100 cumulative reads were also tested using the same prevalence requirement. Library size, observed ASV richness, and Shannon diversity were calculated from the filtered table. Bray–Curtis dissimilarity was calculated from relative abundances and visualized using principal-coordinate analysis (PCoA).

Sequential distance-based permutation tests were used to evaluate sampling age, APS after adjustment for sampling age, and the sampling-age-by-APS interaction [11]. Projection matrices were used to obtain the pseudo-F statistic and R^2^. For each model comparison, R^2^ was calculated as the sum of squares attributable to the tested term divided by the total sum of squares for the samples included in that analysis. Statistical significance was assessed with 4999 permutations in the primary analysis. APS was also tested separately at 30, 60, and 90 days. Corresponding nested linear-model terms for alpha diversity were evaluated with 4999 response permutations. For the ASV-filter sensitivity analysis, each community model was independently refitted with 9999 permutations. Consequently, the 50-read sensitivity run has identical pseudo-F and R^2^ estimates but slightly different Monte Carlo *p* values from the primary 4999-permutation run. Benjamini–Hochberg adjustment [12] was applied within the planned alpha-diversity family and across the six community tests.

ASVs were aggregated at the genus level. Genera were filtered to include only those occurring in at least four samples with a mean relative abundance of at least 0.1%. Counts were log-transformed as log(count + 0.5) and centered within samples. At each sampling age, permutation tests evaluated the dose slope per 100 mg/kg and the mean difference between APS200 and CON. False discovery rates (FDR) were controlled separately within each sampling age and test family. Genus-level results with a nominal *p* < 0.05 but q ≥ 0.05 were considered exploratory.

### 2.3. Intestinal RNA Sequencing and Pathway Analysis

At 90 days, ileal mucosa (archive code HC) and jejunal mucosa (archive code KC) were collected from birds D1–D4, E1–E4, and F1–F4. A sample manifest matched tissue identities and wing numbers to link the cecal content and both mucosal tissues for each bird. Gene-level count and annotation tables were generated under project DZOE2025010368, with 12 libraries per tissue.

Total RNA was extracted using TRIzol reagent (Invitrogen, Carlsbad, CA, USA). RNA purity and integrity were evaluated using a NanoDrop 2000 spectrophotometer (Thermo Fisher Scientific, Waltham, MA, USA) and an Agilent 2100 Bioanalyzer (Agilent Technologies, Inc., Santa Clara, CA, USA), respectively. A260/A280 ratios ranged from 1.99 to 2.08, A260/A230 ratios from 1.95 to 2.36, and RNA integrity numbers (RINs) ranged from 3.0 to 8.4. Following quality review, seven samples with suboptimal integrity but a visible main RNA band were retained for library construction. Sample-level values and treatment-by-tissue summaries are presented in Tables S6 and S7. Libraries were prepared using the VAHTS Universal V10 RNA-seq Library Prep Kit for Illumina (catalog no. NR606; Nanjing Vazyme Biotech Co., Ltd., Nanjing, China). OE Biotech sequenced 150 bp paired-end reads on an NovaSeq 6000 Sequencing System (Illumina, Inc., San Diego, CA, USA). All 24 libraries were included under vendor project DZOE2025010368, with four libraries per diet and tissue. No separate extraction, library-preparation, lane, or sequencing batch variable was present in the archived metadata. Consequently, technical batch was not included as a model term. Raw reads were processed with fastp version 0.23.4 [13] and aligned with HISAT2 version 2.2.1 [14] to the Taihu goose telomere-to-telomere reference genome, Taihu_goose_T2T_genome (BioProject PRJEB75655; NCBI Project ID 1112260) [15]. The corresponding public assembly accessions are RefSeq GCF_040182565.1 and GenBank GCA_040182565.1. Gene-level counts were summarized with HTSeq-count version 2.0.2 [16].

Genes with at least ten counts in four or more samples were retained. Differential expression was estimated separately by tissue using a negative-binomial model in PyDESeq2. The model included treatment as a categorical predictor, CON as the reference group, and positive-count size-factor estimation [17,18]. We summarized results at a threshold of q < 0.05 and at the joint threshold of q < 0.05 and |log_2_ fold change| ≥ 1. Principal component analysis of log_2_(counts per million + 1) was used to visualize sample structure. To evaluate sensitivity to RNA integrity, RIN was standardized within tissue and added as a continuous covariate to the differential expression model. We evaluated log_2_ fold change concordance and compared differentially expressed gene overlap with the primary model. For the principal component analysis, we tested PC1 and PC2 for association with RIN after adjustment for categorical treatment. Additionally, we used a treatment-adjusted permutation test with 9999 permutations restricted within treatment. This test assessed the multivariate contribution of RIN across the 2500 most-variable expressed genes in the transformed expression matrix. These analyses were treated as sensitivity analyses rather than as evidence that low-RIN samples were equivalent to high-RIN samples. For the complete differential expression distributions, genes with non-missing adjusted values were plotted by log_2_ fold change and −log_10_(q). Genes were highlighted only when they met both q < 0.05 and |log_2_ fold change| ≥ 1. Up to five genes per contrast were labeled, prioritizing the smallest q value and then the largest |log_2_ fold change|.

Kyoto Encyclopedia of Genes and Genomes (KEGG) gene sets were constructed from the goose genome annotation. Within each treatment contrast and tissue, genes were ranked by the model Wald statistic and evaluated using preranked gene-set enrichment analysis (GSEA) [19]. Each analysis used 1000 permutations, gene-set sizes ranging from 10 to 500, and a random seed of 20260729. Pathway enrichment was evaluated at q < 0.05 and interpreted from the normalized enrichment score.

### 2.4. Public Ileal Single-Cell Marker Context

Processed single-cell matrices from chicken ileal intraepithelial lymphocytes deposited in the Gene Expression Omnibus (GEO; GSE326338) were used to derive cell identity marker sets [20]. Of the 19,473 downloaded cells, 19,173 passed quality control. CN1 and CN2 processed matrices were byte-identical, so one copy was excluded, and three unique matrices were analyzed. Because the public study examined *Salmonella* Enteritidis infection rather than APS exposure, cell proportions and exposure-related expression effects were not transferred to the goose dataset.

Positive markers were required to have log fold change > 0.25, adjusted *p* < 0.05, and greater detection inside than outside the cluster. The top 150 positive markers from each cluster were grouped by conservative cell identity. For each goose tissue and treatment contrast, genes meeting the joint differential expression threshold were tested for over-representation in each marker set using a one-sided hypergeometric test. The Benjamini–Hochberg adjustment was applied within each goose contrast. This analysis was used solely for cross-species cell-type context.

### 2.5. Wing Number-Linked Paired Multi-Omics Analysis

At 90 days, cecal content, ileal mucosa, and jejunal mucosa were matched by wing number from birds D1–D4, E1–E4, and F1–F4. Accordingly, we used the animal as the unit of paired analysis (12 animals; four per diet). Genera present in at least 6 of the 12 paired animals were ranked by their mean relative abundance. The 18 highest-ranking genera were retained before calculation of the genus–pathway correlations. The seven pathways included butanoate metabolism, glycolysis/gluconeogenesis, oxidative phosphorylation, extracellular-matrix–receptor interaction, Toll-like receptor signaling, mTOR signaling, and Wnt signaling. These pathways spanned fermentation and energy metabolism, epithelial and extracellular-matrix function, innate immune signaling, nutrient sensing, and intestinal homeostasis. The panel was selected after pathway annotation and a broad review of the RNA-seq results, but before the genus–pathway correlation coefficients were calculated. This panel was not preregistered before the RNA-seq analysis. For each tissue, a pathway score was calculated only when at least eight annotated member genes were available. This score was defined as the mean sample-standardized log-counts-per-million value of those genes.

Genus abundances and pathway scores were rank-transformed and residualized against categorical diet. Correlations between the residuals were reported as the diet-adjusted Spearman coefficients. Empirical two-sided *p* values were calculated using 4999 permutations restricted within diet, followed by the Benjamini–Hochberg adjustment across all 252 genus–pathway–tissue tests. These correlations were interpreted as associations and did not establish causal direction.

### 2.6. Reproducibility, Statistical Thresholds, and Figure Production

Primary analyses were conducted using Python 3.12.13, NumPy 2.4.6, pandas 3.0.3, GSEApy 1.1.13, SciPy 1.18.0, scikit-learn 1.9.0, statsmodels 0.14.6, and PyDESeq2 0.5.4, with random seed 20260729. Except for results explicitly designated as exploratory, the multiplicity-adjusted significance threshold was set at q < 0.05. The four animals per diet-by-sampling-age or diet-by-tissue cell limited power for modest effects and interactions. Consequently, the omics component was treated as an exploratory subset rather than a confirmatory, fully powered trial. Input tables were checked for required columns, sample counts, and identifiers during figure preparation. Figures were generated from derived result tables in R version 4.6.1 using repository-relative paths. Vector PDF and SVG files, 600-dpi TIFF files, and panel-level source data were exported. OpenAI Codex Desktop version 26.903.8094.0 (accessed on 9, 16, and 18 September 2026) assisted with code refactoring, consistency checks, and sensitivity analysis implementation. It was also used for R figure code, English-language editing, manuscript formatting, and preparation of the point-by-point response. All computations were executed locally. All tool-assisted outputs were verified against the experimental data and analysis results. The tool did not generate or modify experimental observations. The experimental and analytical hierarchy is summarized in Table 2.

## 3. Results

### 3.1. Cecal Community Structure Varied with Sampling Age and APS

All 36 16S rRNA gene libraries were included in the analysis. The initial dataset contained 4266 ASVs. After taxonomic, abundance, and prevalence filtering, 1524 bacterial ASVs remained. The median filtered library size was 52,837.5 reads.

Sampling age accounted for the largest tested component of the Bray–Curtis variation (pseudo-F = 2.930, R^2^ = 0.1508, *p* = 0.0002, q = 0.0012; Figure 1; Table 3). After adjustment for sampling age, APS explained a smaller component of the variation (pseudo-F = 1.320, R^2^ = 0.0666, *p* = 0.0096, q = 0.0144). The sampling-age-by-APS interaction was significant (pseudo-F = 1.252, R^2^ = 0.1225, *p* = 0.0056, q = 0.0128). Accordingly, the community patterns associated with APS differed across sampling ages under the phase-specific feeding program.

Within-age tests identified the clearest community separation at 60 days (R^2^ = 0.2393, *p* = 0.0064, q = 0.0128). The 30-day association was nominally significant but did not meet the adjusted threshold (R^2^ = 0.2212, *p* = 0.0494, q = 0.0593). At 90 days, the association was not significant (R^2^ = 0.2066, *p* = 0.1190, q = 0.1190; Figure 1c). Neither Shannon diversity nor observed ASV richness was associated with APS after adjustment for age (all treatment q ≥ 0.511). Thus, an APS association was detected for multivariate community composition, whereas no association with alpha diversity met the adjusted significance threshold.

**Table 3:** Cecal community-level permutation tests.

Term	Pseudo-F	R^2^	*p*	q
Sampling age	2.930	0.1508	0.0002	0.0012
APS conditional on sampling age	1.320	0.0666	0.0096	0.0144
Sampling age × APS	1.252	0.1225	0.0056	0.0128
APS at 30 days	1.278	0.2212	0.0494	0.0593
APS at 60 days	1.416	0.2393	0.0064	0.0128
APS at 90 days	1.172	0.2066	0.1190	0.1190

The primary community conclusions were insensitive to the cumulative-read threshold (Table S8). Thresholds of 10, 50, and 100 reads retained 1890, 1524, and 1228 ASVs, respectively. Across these filters, the R^2^ ranges were 0.1506–0.1514 for sampling age, 0.06664–0.06666 for APS conditional on sampling age, and 0.12247–0.12259 for the interaction. The corresponding permutation *p* values were 0.0001, 0.0071–0.0093, and 0.0057–0.0070. The within-age pattern was also stable: treatment *p* values ranged from 0.0476 to 0.0505 at 30 days, from 0.0056 to 0.0069 at 60 days, and from 0.1150 to 0.1320 at 90 days.

### 3.2. No Individual Genus Survived Multiplicity Correction

Fifty-six genera met the analysis criteria. Exploratory dose slope estimates varied across sampling ages (Figure 2). At 30 days, nominal associations involved *Erysipelatoclostridium*, *Anaerofilum*, Prevotellaceae NK3B31 group, and *Parabacteroides.* At 90 days, *Romboutsia* and *Anaerobiospirillum* ranked among the leading nominal associations. However, no genus-level dose slope or APS200-versus-CON contrast met the multiplicity-adjusted significance threshold of q < 0.05. Accordingly, no individual genus was identified as APS-responsive after multiplicity correction.

### 3.3. Intestinal Transcriptional Patterns Varied Across Tissue and Dose Contrasts and Were Sensitive to RIN in Jejunum

After filtering, 13,607 ileal genes and 13,482 jejunal genes were tested. In the ileum, the APS100-versus-CON contrast contained 70 genes with q < 0.05, including 43 with |log_2_ fold change| ≥ 1. In the APS200-versus-CON contrast, six and three genes met the respective criteria (Figure 3; Table 4). The APS100 ileal signature included increased expression of MARCKSL1 and CDH26 and decreased expression of KBTBD12, SH2 domain-containing protein 1B (SH2D1B), and granzyme A (GZMA). The APS200 signature included CD180, PSTPIP2, and CCR9.

In the jejunum, the APS100-versus-CON contrast contained ten genes with q < 0.05, nine of which also met the fold change criterion. The APS200-versus-CON contrast contained seven genes with q < 0.05, all of which met the joint criterion. Representative genes included CDH26, CCL19, and CXCR6 for APS100 and CD180, EOMES, DMBT1, TTPA, and GHR for APS200. The complete gene-level distributions are displayed in Figure S1. The APS100 ileal contrast revealed the broadest stringent response, with 11 increased and 32 decreased genes. The other three contrasts contained three, nine, and seven stringent genes, respectively.

RIN values varied descriptively across treatment-by-tissue cells (Table S7). Mean ± standard deviation (SD) values for CON, APS100, and APS200 were 5.53 ± 1.14, 6.40 ± 2.11, and 4.88 ± 1.10 in the ileum and 3.98 ± 0.62, 5.35 ± 1.88, and 4.03 ± 0.63 in the jejunum, respectively. After adjustment for treatment, the associations of RIN with ileal PC1 (*p* = 0.361) and PC2 (*p* = 0.080) did not reach nominal significance. The multivariate term was also nonsignificant (pseudo-F = 1.246, partial R^2^ = 0.135, *p* = 0.1422). However, in the jejunum, RIN was nominally associated with PC1 (partial R^2^ = 0.544, *p* = 0.0149) and the multivariate expression matrix (pseudo-F = 3.688, partial R^2^ = 0.316, *p* = 0.0093). The PC2 term did not reach nominal significance (*p* = 0.577).

Adding RIN to the negative-binomial model changed the number of genes meeting the joint q and fold change threshold from 43 to 27 (ileal APS100), three to four (ileal APS200), nine to 21 (jejunal APS100), and seven to 14 (jejunal APS200). Genome-wide log_2_ fold change correlations between primary and RIN-adjusted models were 0.944, 0.952, 0.567, and 0.987, respectively. The corresponding overlaps between gene sets meeting the joint threshold were 21, three, three, and seven genes, respectively. Thus, APS200 effect estimates were strongly correlated between models, whereas the jejunal APS100 signature was especially sensitive to RIN adjustment. The primary model results are retained for continuity. However, all transcriptomic findings are interpreted as exploratory and potentially influenced by RNA integrity.

Across the four tissue-by-dose contrasts, 51 pathway–tissue–dose combinations met GSEA q < 0.05 (Figure 4; Table S3). For APS200, oxidative phosphorylation, proteasome, ribosome, tricarboxylic-acid-cycle-related metabolism, and branched-chain amino-acid degradation were negatively enriched in ileum. In the jejunum, oxidative phosphorylation, ribosome, proteasome, tricarboxylic acid cycle, and glycolysis/gluconeogenesis were positively enriched, while extracellular-matrix–receptor interaction and cytoskeletal programs were negatively enriched. For APS100, the jejunum showed positive enrichment of oxidative phosphorylation, ribosomal, cytoskeletal, and extracellular-matrix programs. Furthermore, APS200 gene-level coefficients were weakly correlated between ileum and jejunum (Spearman ρ = 0.07), suggesting different transcriptional patterns across intestinal compartments.

### 3.4. Public Chicken Markers Provided Lymphocyte Context for Part of the Ileal Response

Two marker-set associations met q < 0.05, both in the ileal APS100-versus-CON contrast (Figure 5). Twenty-one stringent goose differentially expressed genes were represented in the public marker universe. Five overlapped with the cytotoxic intraepithelial-lymphocyte set: ALOX5AP, cytokine-dependent hematopoietic cell linker (CLNK), tumor necrosis factor ligand superfamily member 6 (FASLG), GZMA, and SH2D1B (q = 0.000160). Five overlapped with the broader T-lymphocyte set: ADORA2A, CLNK, GZMA, SH2D1B, and XCL1 (q = 0.0125). No marker-set association in the other tissue-by-dose contrasts remained significant after correction. Because the reference dataset was generated in chickens under a different exposure, these overlaps provide cell-type context. However, they do not demonstrate changes in lymphocyte abundance or state in APS-fed geese.

### 3.5. Paired Microbiome–Pathway Associations Did Not Survive Correction

Wing number matching enabled 252 diet-adjusted genus–pathway tests across animals with matched samples at 90 days (Figure 6; Table S4). No association met q < 0.10. The smallest nominal *p* value was observed for the negative association between UCG-005 and the jejunal butanoate metabolism score (ρ = −0.769, restricted permutation *p* = 0.0044, q = 0.8064). The Clostridia UCG-014 group was also nominally associated with this score (ρ = −0.809, *p* = 0.0064, q = 0.8064). Both were retained only as unconfirmed nominal signals.

## 4. Discussion

Dietary APS was associated with variation in cecal community structure across sampling ages within the phase-specific feeding program. APS-associated transcriptional patterns were also observed in the ileum and jejunum. After adjusting for sampling age, APS accounted for 6.7% of Bray–Curtis variation. The strongest within-age separation occurred at 60 days. After multiplicity correction, the significant community-level pattern was not attributable to a single genus. Additionally, none of the paired microbiome–pathway associations remained significant. Consequently, the results support community-level and tissue-specific associations under the study’s feeding program.

The temporal pattern aligns with the known post-hatch development of the goose intestinal microbiota [2,3]. However, chronological age was inseparable from the phase-specific basal diet transition. Birds sampled on day 30 consumed a starter formulation. In contrast, birds sampled on days 60 and 90 consumed a grower formulation from day 31 onward. Accordingly, differences involving day 30 may reflect chronological development, basal diet composition, or both. The day 60 and day 90 groups shared the same basal diet. This shared formulation makes their contrast less directly affected by the day 30 diet switch. However, somatic growth, immune maturation, and environmental exposure still varied with age. Because sampling was cross-sectional, this analysis distinguishes sampling age groups under a specific feeding program. It does not isolate pure chronological age effects or track within-bird microbial trajectories.

No individual genus remained statistically significant after FDR correction. This places the microbial association at the community rather than the taxon level. Community-level tests integrate coordinated but modest variation across multiple taxa. This allows them to detect compositional differences even when no single taxon passes the adjusted threshold. Previous poultry studies reported APS-associated changes in microbial composition and intestinal barrier measurements [5,6]. In addition, several genera described in those studies appeared among the nominal associations in these data. Direct comparison remains limited by differences in species, gut segment, age, health status, APS composition, dose, and analytical workflow. Consequently, the present data support a community-level association without assigning that response to any single genus.

APS-associated transcriptional patterns were observed across the dose-by-tissue contrasts. The larger number of stringent genes for APS100 than for APS200 does not establish a stronger physiological effect at the lower dose. Statistical detection also depends on dispersion, baseline expression, RNA integrity, and overall effect size. Several energy- and matrix-related pathways showed opposite enrichment directions between tissues, and gene-level coefficients were only weakly correlated. These exploratory patterns support evaluating intestinal compartments separately; formal tests of treatment-by-tissue interaction and independent validation are needed to establish differences in treatment effects between tissues. Specifically, RIN was associated with the jejunal expression configuration after treatment adjustment. The jejunal APS100 effect estimates and the set of genes meeting the joint significance and fold change thresholds were particularly sensitive to RIN adjustment. The primary jejunal signatures may reflect both treatment-related and RNA-quality-related variation. RIN-adjusted analyses are reported as sensitivity checks and do not replace independent molecular validation.

Overlap between the APS100 ileal signature and T-lymphocyte and cytotoxic intraepithelial-lymphocyte markers is consistent with the immunoregulatory literature on APS [5]. However, the reference dataset profiled chicken ileal lymphocytes after *S.* Enteritidis infection [20]. Because of this, cross-species marker overlap cannot establish that APS altered lymphocyte abundance or cell state in geese. That interpretation requires direct validation in the goose intestine using flow cytometry, immunohistochemistry, or replicated single-cell profiling.

Wing number matching preserved within-animal correspondence, while diet residualization and within-diet permutations reduced correlations attributable to treatment-group structure. No paired association survived correction. With four animals per diet, heterogeneous pathway scores, and no direct measurement of microbial metabolites, the analysis was not designed to establish mediation. Larger paired cohorts, a prospectively defined metabolite-informed panel, direct measurements of short-chain fatty acids and bile acids, and temporal ordering of microbial and host responses will be required for that purpose.

Several limitations define the scope of inference. First, only four animals were sequenced per diet-by-sampling-age or diet-by-tissue cell. This small sample size limited the statistical power to detect modest effects and interactions. It also prevented reliable estimation of cage-, sex-, or broader individual-level heterogeneity. Second, key technical metadata were unavailable. Although the feeding trial used five replicate cages per diet and balanced sex within each cage, the direct cage identifiers and sex of the sequenced animals were not retained. Consequently, cage and sex could not be included in the omics models, and possible within-cage non-independence remains an unquantified limitation. Similarly, the archived technical metadata did not permit a cage-by-sequencing-batch assessment. Third, selection of birds near the cage mean may have narrowed body weight variation. Furthermore, linked individual body weights and raw cage-level or sex-stratified growth records were not available for valid correlation or performance reanalysis. Several RNA samples exhibited low integrity, and no independently validated RT-qPCR dataset was available for the archived experiment. The commercial APS preparation was characterized by a supplier-reported polysaccharide content threshold rather than a lot-specific chemical fingerprint or molecular-weight distribution. The two supplemented levels cannot define a complete dose–response curve or identify an optimum dose. The 16S rRNA gene profiles were cross-sectional. Absolute bacterial load, metabolomics, and shotgun functional profiles were unavailable, and the public single-cell reference differed in species and exposure. These constraints require the findings to be interpreted as exploratory treatment-associated patterns within the documented cohort.

Future experiments should use chemically characterized APS and larger omics cohorts with explicit cage-balanced and sex-recorded sampling. To distinguish chronological development from feeding-phase effects, future studies should evaluate age and basal diet formulation in a factorial design, using separate animals for terminal cecal and intestinal sampling at each planned age. At each endpoint, specimens collected from a given bird should remain linked to its body weight and cage records. RT-qPCR, absolute microbial load, metagenomics, short-chain fatty acids, bile acids, epithelial morphology, and immune-cell measurements would then permit direct validation of the tissue-specific transcriptional and community patterns identified here.

## 5. Conclusions

Dietary APS was associated with cecal community structure across sampling ages within the phase-specific feeding program in Taihu geese, with the clearest separation at 60 days. At 90 days, exploratory APS-associated transcriptional patterns differed across tissue and dose contrasts and were sensitive to RNA integrity, particularly in the jejunal APS100 contrast. Public single-cell markers linked part of the APS100 ileal signature to lymphocyte-related genes. No individual genus or paired genus–pathway association survived false discovery rate correction. These exploratory results characterize temporal and intestinal-compartment associations with APS without assigning the temporal pattern solely to chronological age; the inability to assess within-cage dependence further limits inference.

## Figures and Tables

**Figure 1:**
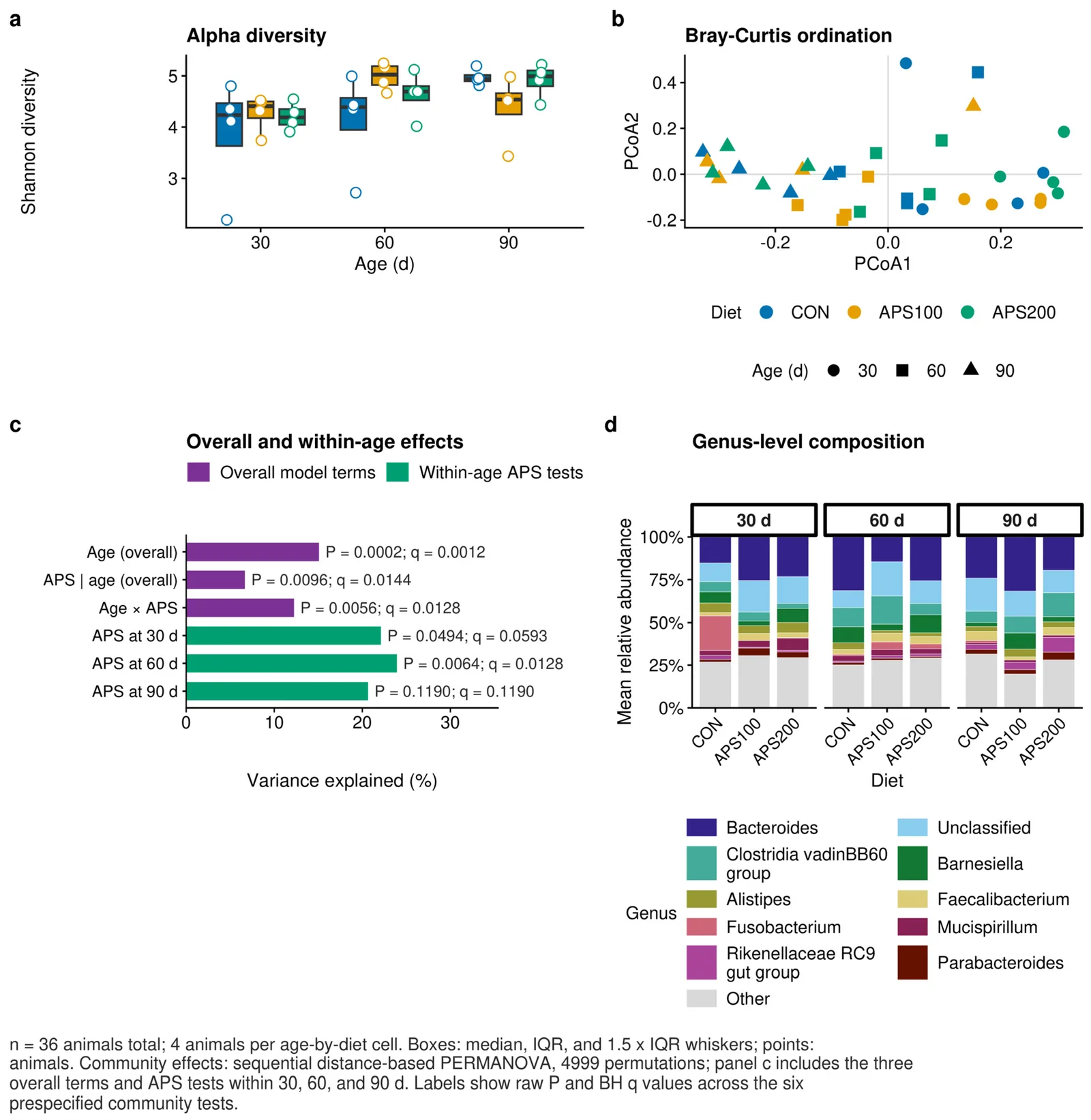
Cecal community structure across sampling ages and APS treatments. (**a**) Shannon diversity by sampling age and diet. Points represent individual animals (n = 4 per diet and sampling age). (**b**) Bray–Curtis principal coordinate analysis. Colors denote diets and symbols denote sampling age (36 libraries). (**c**) Proportion of total variation explained by the three overall sequential PERMANOVA terms and by APS tested separately within 30, 60, and 90 days. Labels report raw permutation *p* values and Benjamini–Hochberg-adjusted q values. (**d**) Mean genus-level relative abundance by diet and sampling age. Community-level tests used 4999 permutations. Benjamini–Hochberg adjustment was applied across the six planned tests.

**Figure 2:**
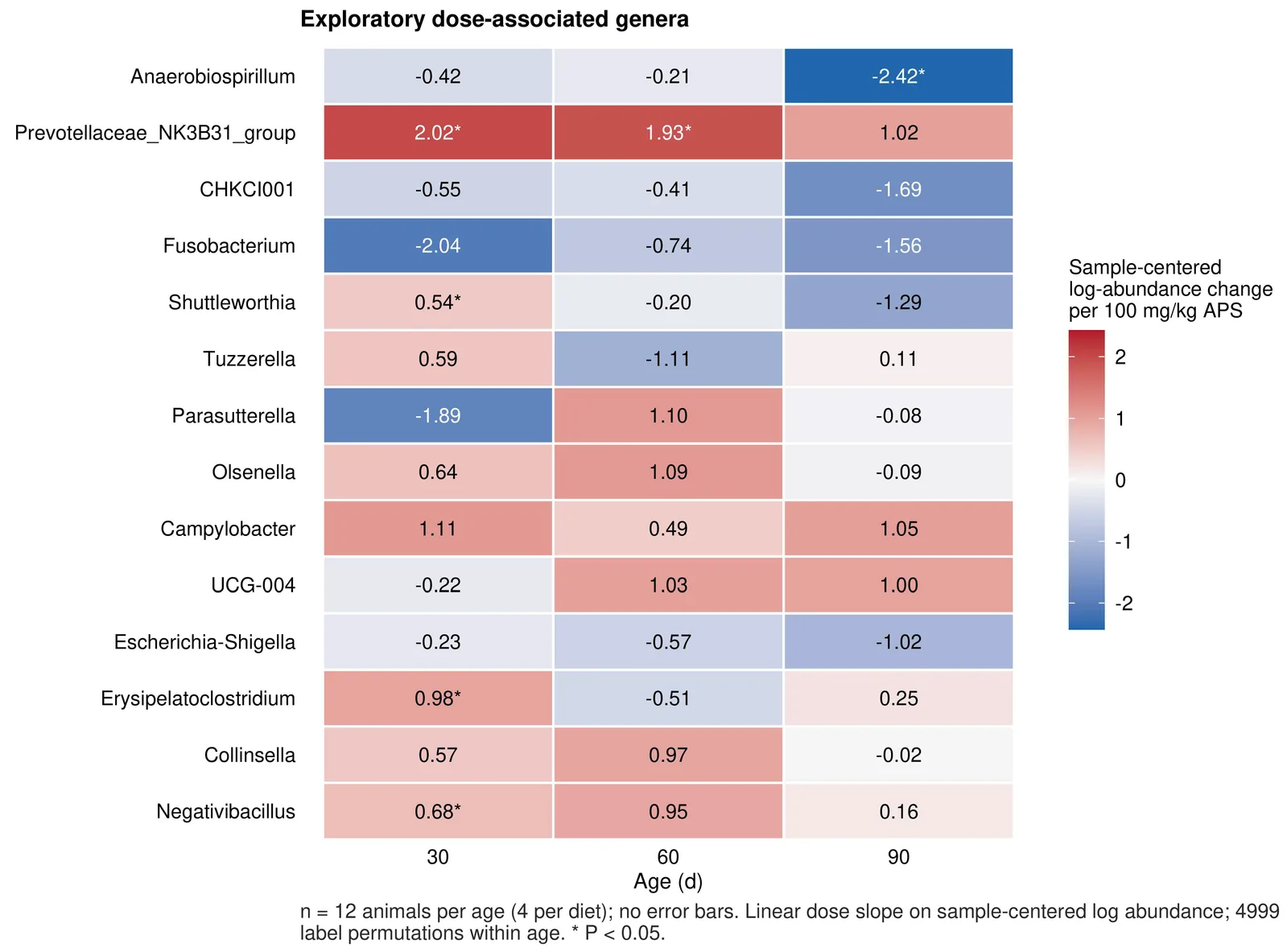
Exploratory genus-level dose slopes. Sample-centered log-abundance change per 100 mg/kg APS is shown by sampling age for the leading predefined ranking set (n = 4 animals per diet and sampling age). Symbols denote nominal permutation *p* < 0.05. No tested genus survived Benjamini–Hochberg adjustment within its sampling age and test family. Estimates are exploratory and describe changes on the sample-centered log(count + 0.5) scale; they do not estimate absolute abundance changes.

**Figure 3:**
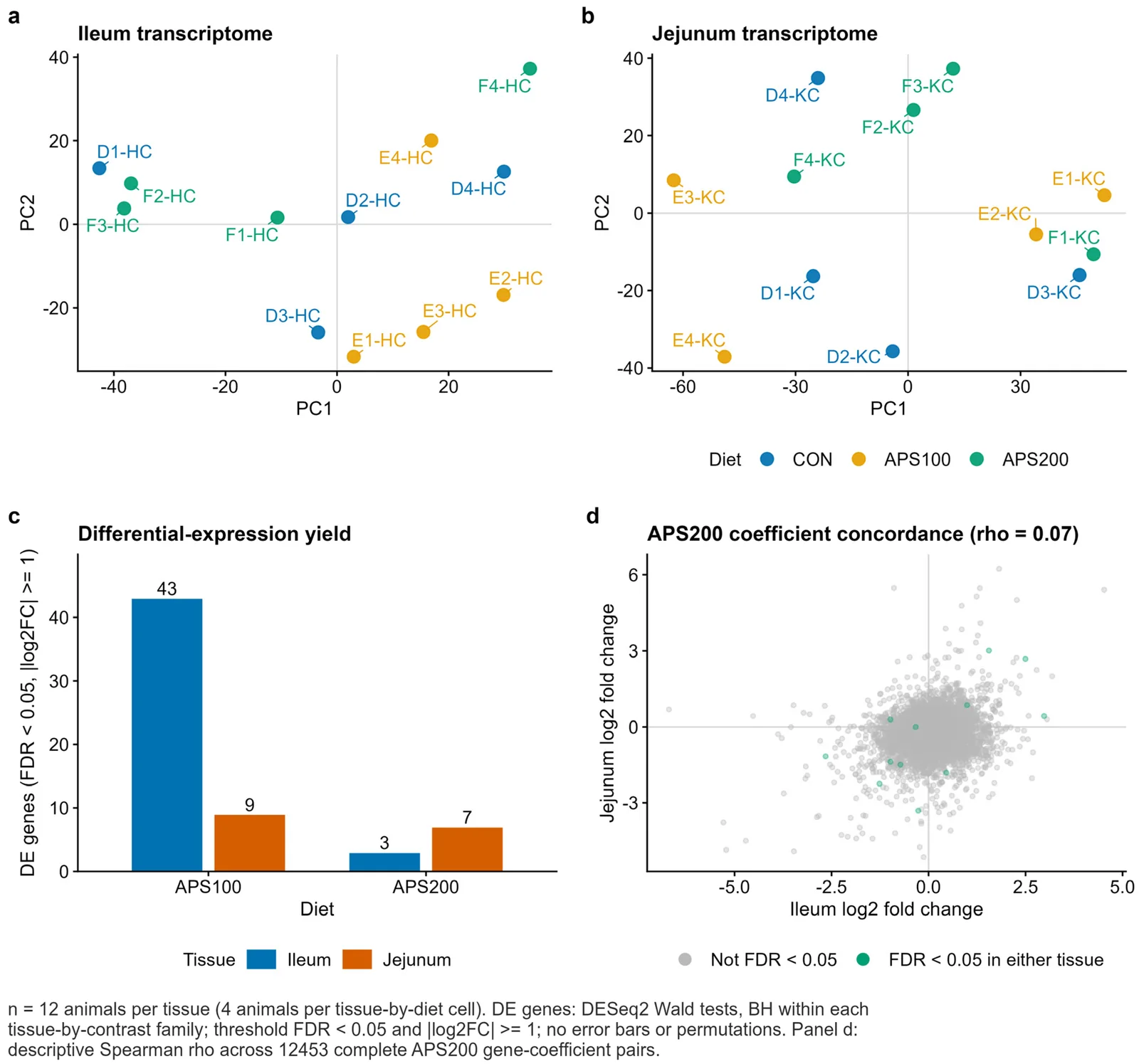
Intestinal transcriptional patterns across tissue and dose contrasts. (**a**,**b**) Principal component maps of ileal and jejunal RNA-seq libraries (n = 4 per diet and tissue). (**c**) Numbers of genes meeting q < 0.05 and |log_2_ fold change| ≥ 1 in each APS-versus-CON contrast. (**d**) Cross-tissue concordance of APS200 gene-level coefficients. Highlighted genes had q < 0.05 in at least one tissue. Differential expression was estimated separately by tissue using a negative-binomial model and Benjamini–Hochberg adjustment.

**Figure 4:**
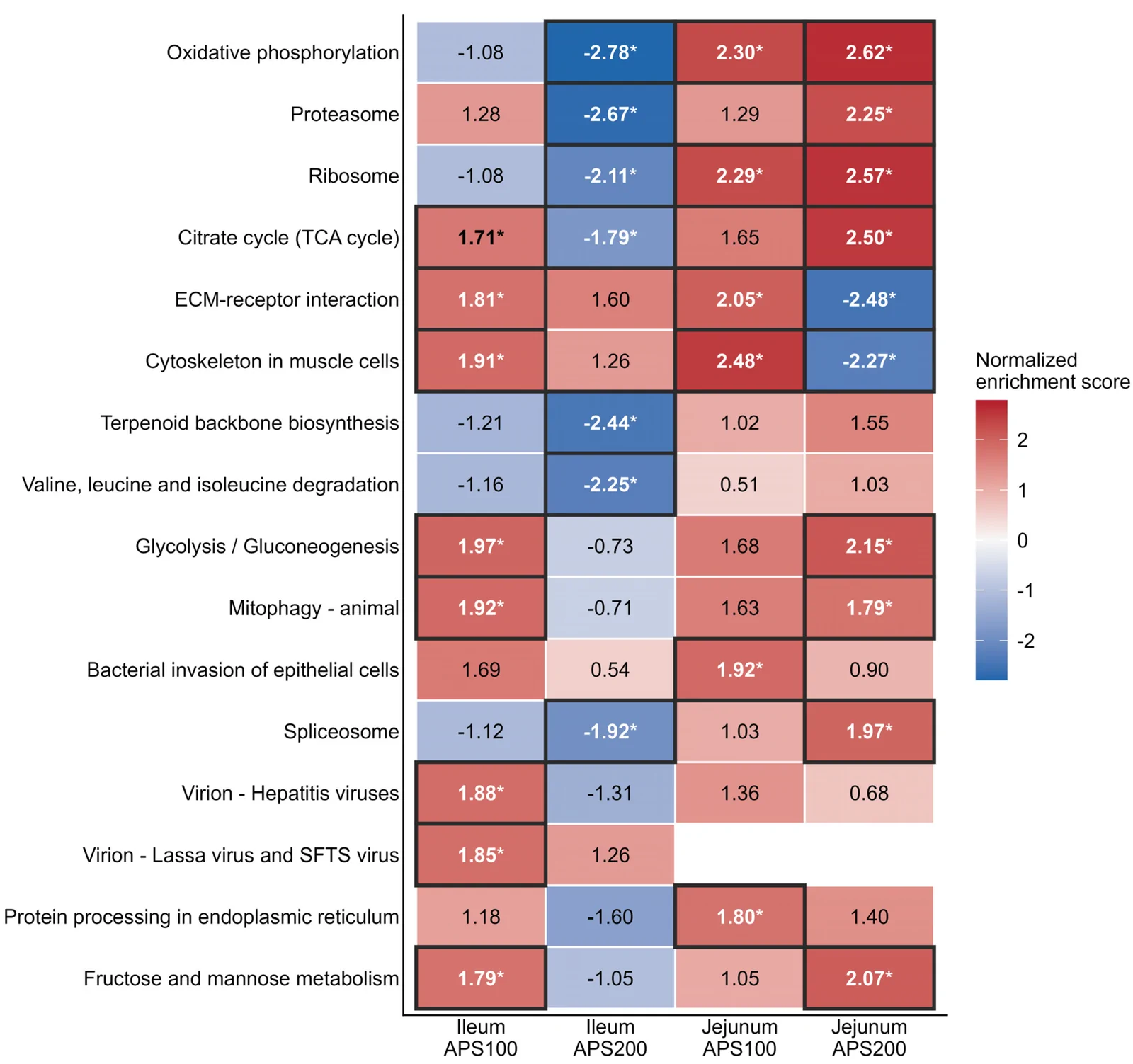
KEGG preranked gene-set enrichment. Normalized enrichment scores are shown for leading pathway–tissue–dose combinations. An asterisk (*) and a dark cell outline indicate pathways with a GSEA FDR q-value < 0.05; direction is relative to CON within tissue. Blank cells indicate unavailable enrichment results, not zero enrichment. Gene sets contained 10–500 annotated genes, and enrichment used 1000 permutations.

**Figure 5:**
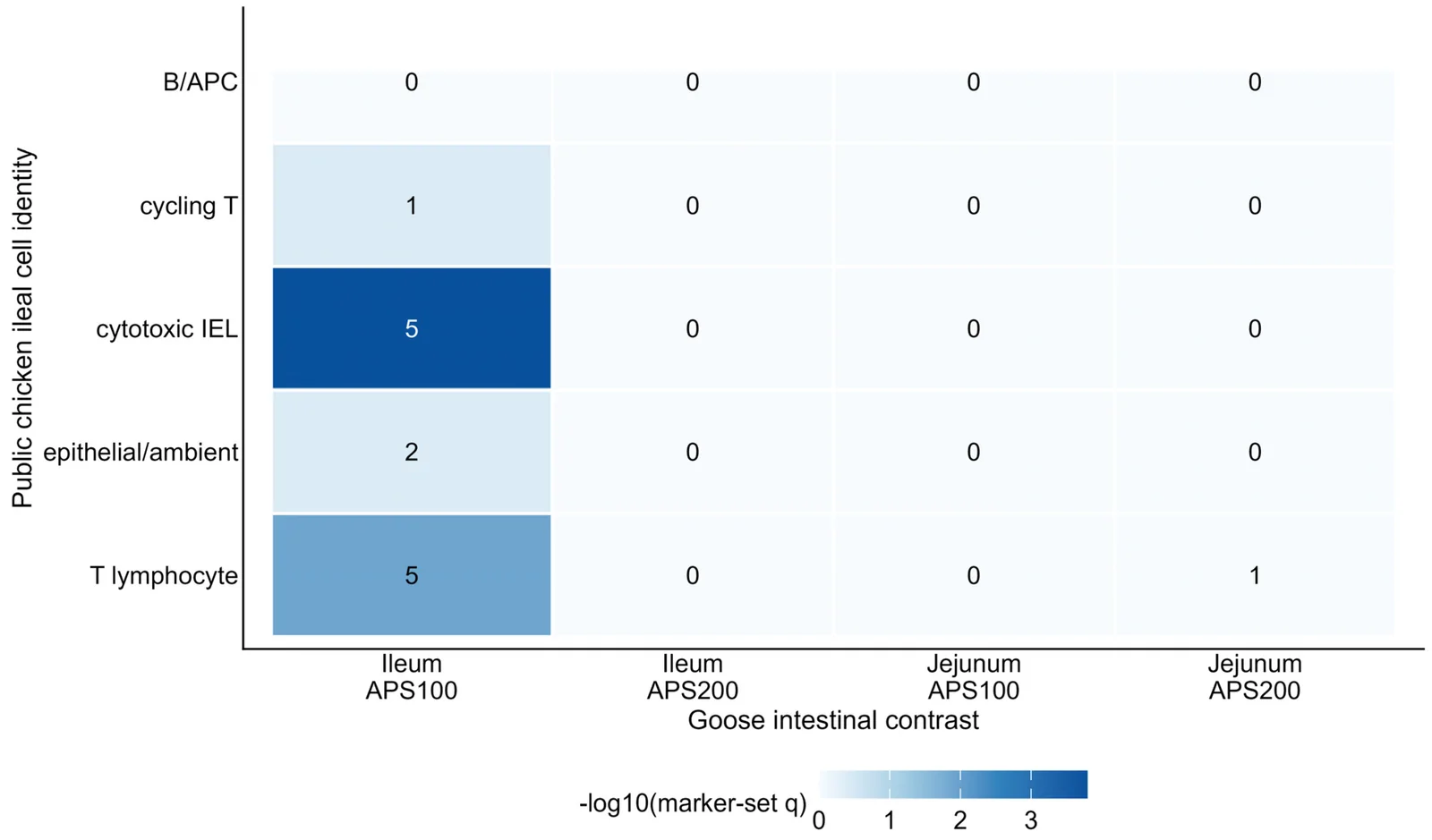
Public chicken ileal cell marker context for goose differentially expressed genes. Color denotes −log_10_ marker-set q, and cell labels give the number of overlapping stringent goose differentially expressed genes. Hypergeometric tests were adjusted within each goose tissue-by-dose contrast. The public chicken marker sets were derived from GSE326338. Infection effects and cell fractions were not transferred to the goose experiment.

**Figure 6:**
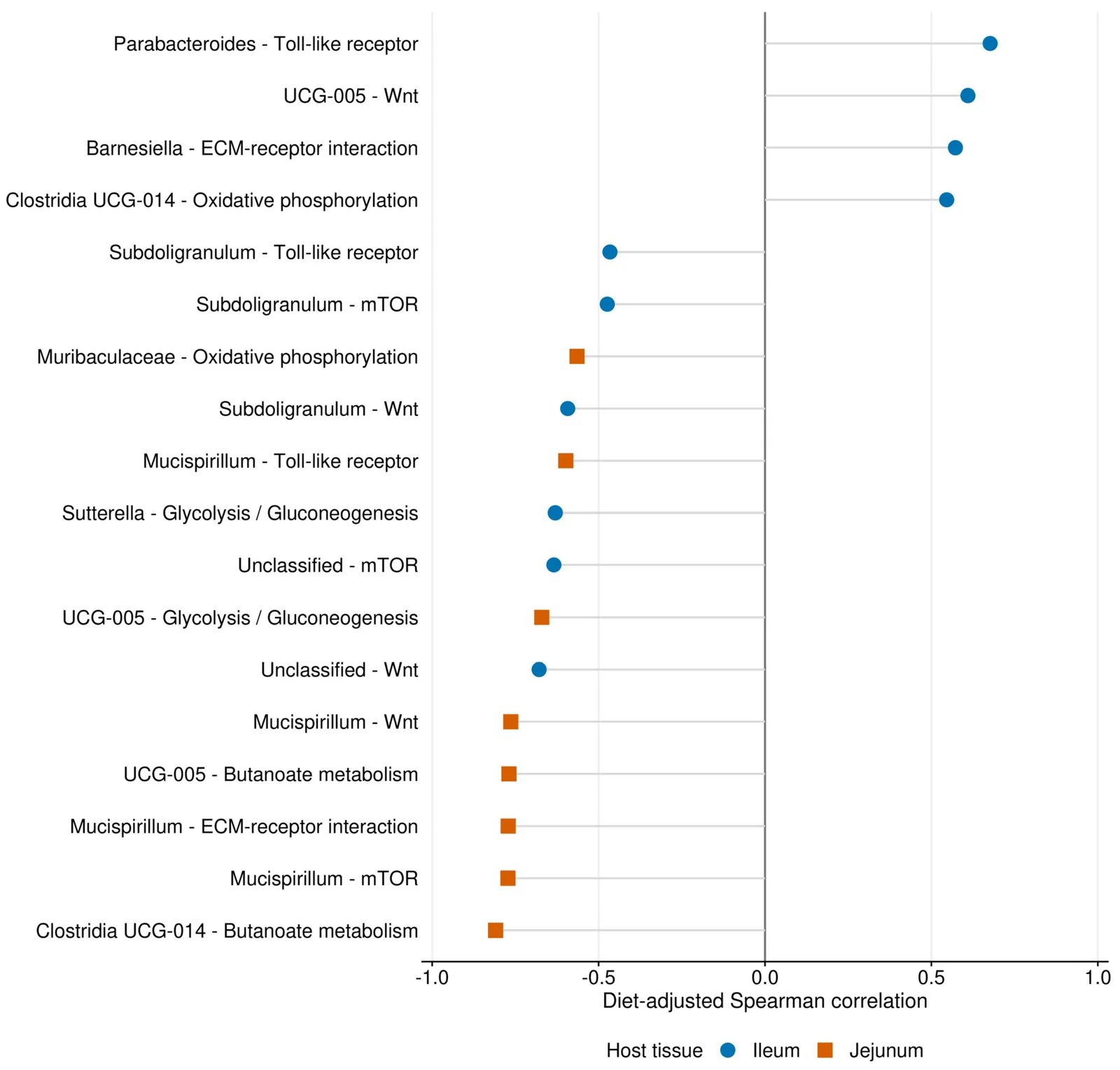
Exploratory paired microbiome–host pathway associations at 90 days. Leading diet-adjusted rank correlations across animals with matched samples are shown for ileal and jejunal pathway scores (12 matched animals; n = 4 per diet). Two-sided empirical *p* values used 4999 permutations restricted within diet, with Benjamini–Hochberg adjustment across all 252 genus–pathway–tissue tests. No association passed q < 0.10.

**Table 1:** Basal diet ingredients and selected calculated and analyzed nutrient levels.

Item	Days 1–30	Days 31–90	Nutrient Level	Days 1–30	Days 31–90
Corn, %	60.00	61.00	Metabolizable energy, MJ/kg	11.01	11.48
Soybean meal, %	22.00	17.00	Crude protein, %	15.33	13.57
Wheat bran, %	7.50	0.00	Ether extract, %	2.55	2.45
Rice bran, %	2.71	14.00	Crude fiber, %	5.50	7.00
Calcium carbonate, %	1.90	3.00	Calcium, %	0.815	0.933
Sodium bicarbonate, %	0.80	0.00	Total phosphorus, %	0.60	0.35
Methionine, %	0.09	0.00	Methionine, %	0.32	0.19
Goose premix, %	5.00	5.00	Lysine, %	1.02	0.76
Total, %	100.00	100.00	Sodium chloride, %	0.35	0.35

Note: Ingredient proportions, metabolizable energy, ether extract, and sodium chloride are formulation-based values. Crude protein, crude fiber, calcium, total phosphorus, methionine, and lysine are analyzed values for retained diet samples 802 (report A01C2600677; sample C260567) and YH (report A01C2600675; sample C260566), respectively. The reporting basis was not specified in the laboratory reports; analyzed values are presented as reported, without conversion between as-fed and dry-matter bases. At an inclusion rate of 5%, the goose premix was formulated to supply the following amounts per kilogram of complete diet: vitamin A, 195,000 IU; vitamin D, 60,000 IU; vitamin E, 600 IU; vitamin K, 30 mg; vitamin B_1_, 20 mg; vitamin B_2_, 100 mg; calcium pantothenate, 130 mg; niacinamide, 400 mg; vitamin B_6_, 50 mg; folic acid, 15 mg; vitamin B_12_, 300 μg; Fe, 2000 mg; Cu, 2000 mg; Zn, 2000 mg; I, 15 mg; and Se, 6 mg. These formulation-based amounts represent the contribution from the premix only and exclude nutrients supplied by the other feed ingredients.

**Table 2:** Experimental and analytical design.

Data Layer	Design and Observational Unit	Primary Analysis
Feeding trial	3 diets × 5 replicate cages × 10 birds = 150 birds; each cage contained 5 males and 5 females	Cage was the experimental unit for the feeding trial
Cecal 16S rRNA	3 diets × 3 sampling ages × 4 animals	Animal was the observational unit. Direct cage and sex identifiers were unavailable
Intestinal RNA-seq	2 tissues; 3 diets × 4 animals at 90 days	Animal was the observational unit. Primary and RIN-adjusted differential expression and KEGG GSEA
Paired multi-omics	12 animals linked by wing number	Diet-adjusted correlations with within-diet permutations
Public marker context	GSE326338; 3 unique processed single-cell matrices	Marker-set over-representation only

**Table 4:** Differential expression yield.

Tissue	Contrast	Genes Tested	q < 0.05	q < 0.05 and |log_2_FC| ≥ 1
Ileum	APS100 versus CON	13,607	70	43
Ileum	APS200 versus CON	13,607	6	3
Jejunum	APS100 versus CON	13,482	10	9
Jejunum	APS200 versus CON	13,482	7	7

## Data Availability

The 36 raw cecal 16S rRNA gene amplicon datasets are publicly available in the NCBI Sequence Read Archive under BioProject PRJNA1504520 (SRA study SRP722618; runs SRR39911570–SRR39911605). The 24 raw ileal and jejunal RNA-seq datasets are deposited in NCBI under BioProject PRJNA1504892. Processed chicken ileal single-cell data used for marker context are available from GEO under accession GSE326338. The Supplementary files provide processed data tables and sensitivity analysis results. Analysis scripts retained in the project archive are available from the corresponding authors upon reasonable request.

## Supplementary Materials

The following supporting information can be downloaded at: https://www.mdpi.com/article/10.3390/microorganisms14102149/s1, Supplementary Methods S1: Head-Only Electrical Stunning, Neck Exsanguination, and Confirmation of Death in Geese; Figure S1: Gene-level intestinal transcriptome responses to APS; Table S1: Genus relative-abundance matrix; Table S2: Complete differential-expression results; Table S3: All KEGG preranked enrichment results; Table S4: All paired microbiome–pathway tests; Table S5: Omics sample manifest and accession mapping; Table S6: Sample-level RNA quality-control values; Table S7: RIN summaries and RNA-seq sensitivity analyses; and Table S8: ASV-filter threshold sensitivity analyses. Panel-level figure source data, the configurable analysis entry point, input preflight, reviewer-sensitivity script, and R figure-generation script are also included.

## Abbreviations

The following abbreviations are used in this manuscript:

APS	Astragalus polysaccharides
ASV	Amplicon sequence variant
FDR	False discovery rate
GEO	Gene Expression Omnibus
GSEA	Gene-set enrichment analysis
KEGG	Kyoto Encyclopedia of Genes and Genomes
PCoA	Principal-coordinate analysis
