# Supplementary material for: Sampling-Age-Associated Cecal Community and Intestinal Transcriptional Responses to Dietary Astragalus Polysaccharides in Taihu Geese
Source: Microorganisms. 2026 Sep 23;14(10):2149. doi: 10.3390/microorganisms14102149 (PMC13707507; doi:10.3390/microorganisms14102149)
Supplement: Supplementary file 1 [file microorganisms-14-02149-s001.zip › Supplementary_Materials.pdf]

Supplementary Materials

Sampling-Age-Associated Cecal Community and Intestinal Transcriptional Responses to Dietary Astragalus Polysaccharides in Taihu Geese

Manuscript ID: microorganisms-4515315

Contents: Supplementary Methods S1; Supplementary Figure S1; and an inventory of Tables S1–S8, which are supplied as separate files.

Supplementary Methods S1

Head-Only Electrical Stunning, Neck Exsanguination, and Confirmation of Death in Geese

All procedures followed the institutional *Standard Operating Procedure for Head-Only Electrical Stunning, Neck Exsanguination, and Confirmation of Death in Geese* (JSAHVC-SOP-GOOSE-001, Version 1.0). Personnel performing restraint, stunning, neck cutting, monitoring, and tissue collection had completed training in animal handling and poultry stunning.

Restraint, equipment checks, and electrode placement

Each goose was handled individually and placed in a bleeding cone appropriate for its body size. A constant-current handheld poultry stunner was used for head-only electrical stunning. Clamp electrodes were positioned bilaterally over the region extending from immediately behind the eyes to immediately rostral of the external ear openings so that the current traversed the brain. When necessary, the electrode contact sites were moistened with a small amount of 0.9% sodium chloride solution to reduce contact impedance. Current was applied only after complete and stable electrode contact had been established.

The stunner displayed or recorded current, voltage, and application time. Before each sampling session, trained personnel checked device function in accordance with the manufacturer's instructions. Calibration and maintenance were performed at the prescribed intervals. The equipment manufacturer, model, device identifier, calibration date, and actual current, voltage, and application time for each goose were documented in the procedural record.

Electrical stunning and assessment of unconsciousness

Head-only stunning was delivered at 600 mA root mean square using 50 Hz sinusoidal alternating current for at least 4 s per bird. Stunning effectiveness was assessed immediately after current cessation. Evidence of an effective stun included tonic–clonic activity, loss of posture when observable, absence of spontaneous or rhythmic breathing, and absence of corneal and palpebral reflexes.

Any rhythmic breathing, spontaneous blinking, positive corneal or palpebral reflex, vocalization, righting response, active swallowing, head shaking, or head lifting was considered evidence of an ineffective stun or recovery of consciousness. If any such sign was observed, the goose was immediately restunned using an independently function-checked backup stunner.

Neck cutting and rescue procedure

Neck cutting was performed only after unconsciousness had been confirmed. A second trained operator used a sharp, dedicated knife to make one continuous ventral neck cut that completely severed both carotid arteries and both jugular veins. The target interval from cessation of electrical current to completion of the neck cut was no more than 5 s and was never to exceed 10 s.

If the first stun was ineffective or any sign of recovery occurred during exsanguination, immediate restunning was required. If the stunning equipment failed, sampling was stopped, the ethics-committee-approved backup killing procedure was initiated, and the electrical output, electrode contact, and operating sequence were checked. Sampling resumed only after the fault had been corrected and normal function confirmed. Repeat stunning, equipment abnormalities, and deviations from the SOP were documented.

Observation and confirmation of death

After neck cutting, each goose remained in the bleeding cone under continuous observation for at least 2 min. Death was confirmed only when all of the following criteria were satisfied: absence of spontaneous breathing; absence of detectable heartbeat or heart sounds for 60 s; absence of corneal and palpebral reflexes; no response to firm toe pinch; fixed and dilated pupils; and complete generalized muscle relaxation. Blood and tissue collection, dissection, and any other carcass manipulation began only after the 2-min observation period and confirmation that every death criterion had been fulfilled. No potentially painful procedure was performed before death had been confirmed.

Supplementary Figure S1

Gene-level intestinal transcriptome responses to APS

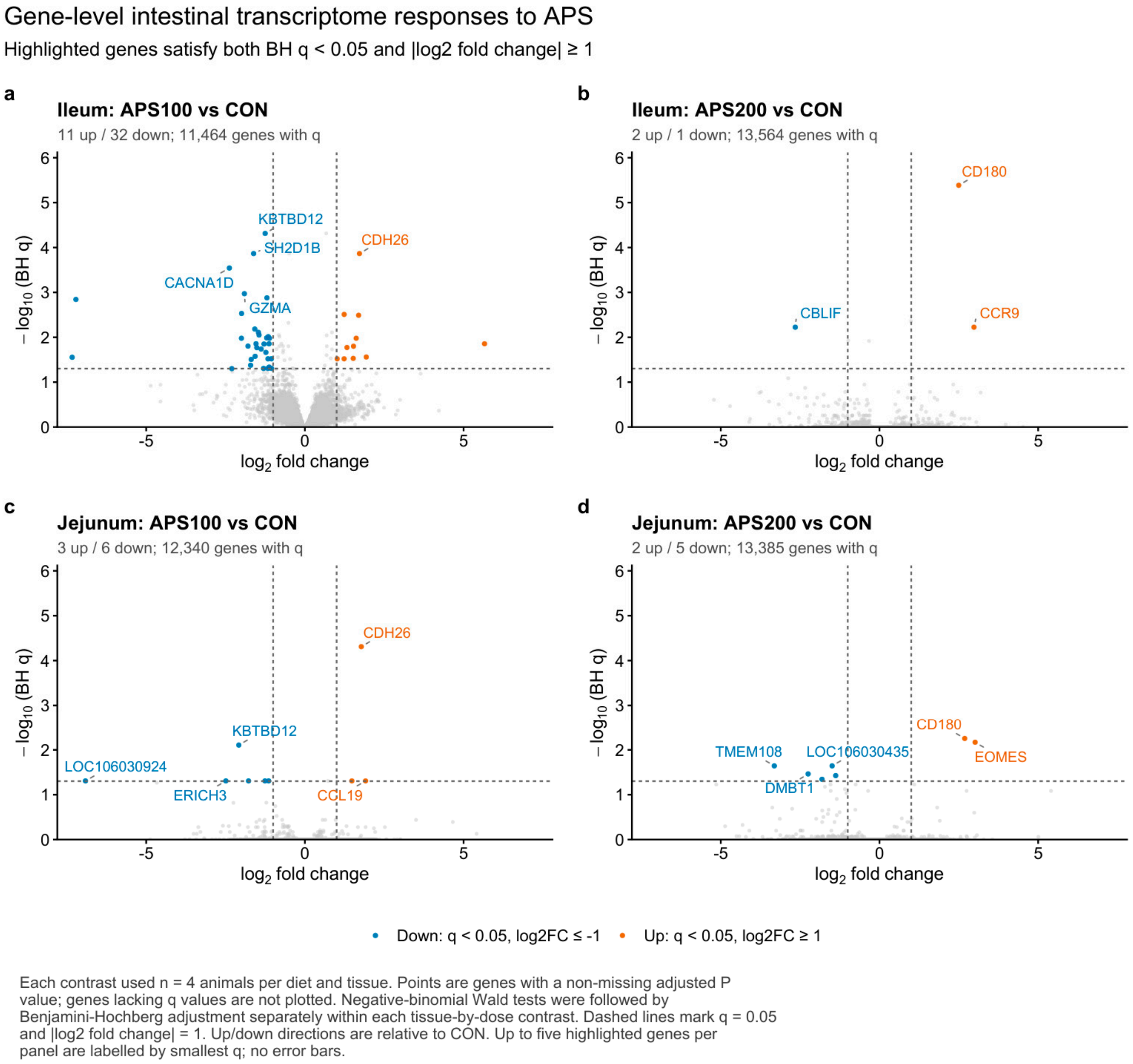

Supplementary Figure S1. Gene-level intestinal transcriptome responses to APS. Volcano plots show $\log_2$ fold change against $-\log_{10}$ Benjamini–Hochberg-adjusted q for (a) ileum, APS100 versus CON; (b) ileum, APS200 versus CON; (c) jejunum, APS100 versus CON; and (d) jejunum, APS200 versus CON. Each contrast included n = 4 animals per diet and tissue. Points represent genes with a non-missing adjusted value. The Figure was generated in R 4.6.1. Blue and orange points indicate genes meeting both $q < 0.05$ and $|\log_2 \text{fold change}| \geq 1$, with direction defined relative to CON; grey points do not meet both criteria. Dashed lines mark $q = 0.05$ and $\log_2$ fold change $= \pm 1$. Negative-binomial Wald tests were followed by Benjamini–Hochberg adjustment separately within each tissue-by-dose contrast. Up to five highlighted genes per panel are labelled by smallest q; no error bars are shown.

Supplementary Tables S1–S8

Tables S1–S8 are supplied as separate files and are not duplicated in this combined document.

Table	Separate file	Contents
**Table S1**	Table_S1_genus_relative_abundance.csv	Genus relative-abundance matrix
**Table S2**	Table_S2_complete_DESeq2_results.xlsx	Complete differential-expression results
**Table S3**	Table_S3_all_KEGG_GSEA.csv	All KEGG preranked enrichment results
**Table S4**	Table_S4_paired_microbiome_pathway_associations.csv	All paired microbiome–pathway tests
**Table S5**	Table_S5_omics_sample_manifest.csv	Omics sample manifest and accession mapping
**Table S6**	Table_S6_RNA_sample_QC.csv	Sample-level RNA quality-control values
**Table S7**	Table_S7_RNA_RIN_and_robustness.xlsx	RIN summaries and RNA-seq sensitivity analyses
**Table S8**	Table_S8_ASV_threshold_sensitivity.xlsx	ASV-filter threshold sensitivity analyses
